# The outcomes of treatment for homebound adults with complex medical conditions in a hospital-at-home unit in the southern district of Israel

**DOI:** 10.1186/s13584-024-00595-y

**Published:** 2024-02-14

**Authors:** Boris Punchik, Ilona Kolushev-Ivshin, Ella Kagan, Ella Lerner, Natalia Velikiy, Suzann Marciano, Tamar Freud, Rachel Golan, Ella Cohn-Schwartz, Yan Press

**Affiliations:** 1 Geriatric Unit, The Haim Doron Division of Community Health, Faculty of Health Sciences, Ben-Gurion University of the Negev, PO Box 653, 84105 Beer-Sheva, Israel; 2 Home Care Unit, Clalit Health Services, South District, Beer-Sheva, Israel; 3 Siaal Research Center for Family Medicine and Primary Care, The Haim Doron Division of Community Health, Faculty of Health Sciences, Ben-Gurion University of the Negev, Beer-Sheva, Israel; 4 Department of Epidemiology, Biostatistics and Community Health Science, Faculty of Health Sciences, Ben-Gurion University of the Negev, Beer-Sheva, Israel; 5 Department of Geriatrics, Soroka Medical Center, Beer-Sheva, Israel; 6 Center for Multidisciplinary Research in Aging, Ben-Gurion University of the Negev, Beer-Sheva, Israel

**Keywords:** Hospital-at-home, Homebound patients, Readmission, Elder patients, Geriatric assessment

## Abstract

**Background:**

A model of hospital-at-home services called the Home Care Unit (“the unit”) has been implemented in the southern region of the Clalit Healthcare Services in Israel. The aim of the present study was to characterize this service model.

**Methods:**

A retrospective cross-over study. included homebound patients 65 years of age and above who were treated for at least one month in the framework of the unit, between 2013 and 2020. We compared the hospitalization rate, the number of hospital days, the number of emergency room visits, and the cost of hospitalization for the six-month period prior to admission to the unit, the period of treatment in the unit, and the six-month period following discharge from the unit.

**Results:**

The study included 623 patients with a mean age of 83.7 ± 9.2 years with a mean Mini-mental State Examination (MMSE) score of 12.0 ± 10.2, a mean Charlson Comorbidity Index (CCI) of 3.7 ± 2.2 and a Barthel Index score of 23.9 ± 25.1. The main indications for admission to the unit were various geriatric syndromes (56.7%), acute functional decline (21.2%), and heart failure (12%). 22.8% died during the treatment period and 63.4% were discharged to ongoing treatment by their family doctor after their condition stabilized. Compared to the six months prior to admission to the unit there was a significant decrease (per patient per month) in the treatment period in the number of days of hospitalization (2.84 ± 4.35 vs. 1.7 ± 3.8 days, p < 0.001) and in the cost of hospitalization (1606 ± 2170 vs. 1066 ± 2082 USD, p < 0.001).

**Conclusions:**

Treatment of homebound adults with a high disease burden in the setting of a hospital-at-home unit can significantly reduce the number of hospital days and the cost of hospitalization. This model of service for homebound patients with multiple medical problems maintained a high level of care while reducing costs. The results support the widespread adoption of this service in the community to enable the healthcare system to respond to the growing population of elderly patients with medical complexity.

## Introduction

A high rate of emergency room visits, recurrent hospitalizations, and increased healthcare costs are a common problem among older patients [1–3]. One of the solutions to this problem is is a hospital-at-home service [4–6]. Interventions in the hospital-at-home service usually include adjusting medications, intravenous treatment, care for complex sores, pain control, the use of various feeding methods, home rehabilitation, and end of life support therapy as in terminal cancer or dementia. The most investigated indications for the hospital-at-home service are specific conditions such as exacerbation of Chronic Obstructive Pulmonary Disease (COPD) or heart failure, acute skin infection, or pneumonia [7–12]. The treatment of these conditions through the hospital-at-home service leads to a reduction in the rate of emergency room visits and repeated hospitalizations and reduction of costs [13–15]. At the same time, a minority of studies included homebound older patients with multiple co-morbidity and geriatric syndromes and yielded conflicting results [4, 16–18] so the actual benefit of hospital-at-home service in this population is not sufficiently clear. The National Health Insurance in Israel provides health services, including for this population, through Health Maintenance Organizations (HMO’s). Clalit Health Services (CHS), as the largest HMO in Israel, has established various models of hospital-at-home service in recent years. In 2013, two hospital-at-home units for homebound patients (“the units”) were established in two large cities in southern Israel, Beer-Sheva and Ashkelon. They included a multidisciplinary team of geriatricians and registered geriatric nurses, physical and occupational therapists, dieticians, social workers, and office services. The staff was available from 8:00 a.m. to 4:00 p.m. five days a week for planned and urgent home visits and took full responsibility for treatment, instead of the medical staff of the primary care clinic. The admission criteria included repeat hospitalizations, complex medical conditions such as severe heart failure (grade 3–4 by New York Heart Associations) [19], stage 3–4 pressure ulcers, geriatric syndromes such as dementia with behavioral and psychological symptoms (BPSD), malnutrition, polypharmacy, a new feeding tube, or palliative care for patients with advanced dementia based on accepted criteria [20]. Most of the patients were referred to the service by family doctors from primary care clinics, while others were referred by the hospital medical staff. Admission to the service was contingent upon the patient’s or a proxy’s consent to receive hospital-at-home treatment, and the presence of family members or a nursing aide at the bedside throughout the day. At the first home visit the unit team conducted a comprehensive geriatric assessment, including assessment of physical, cognitive, functional, and nutritional status, ambulation, home safety, and auxiliary nursing evaluations such as pain assessment, diabetic foot, et al. Following the first visit a comprehensive intervention plan was developed that included regular home visits (by a geriatrician and a nurse), supplemental visits by a dietician, a social worker, a physical therapist, and an occupational therapist, and a telephone assistance line available to patients and their family members. All staff members could review the patient’s medical records and all visit summaries are automatically uploaded to the primary care clinic. The decision to discharge a patient was reached by the multidisciplinary team at the periodic staff meeting after the patient reached his/her treatment goals. There was no cost to the patient for treatment. All team personnel were employed by the CHS and did this work as part of their job.

The aims of the present study were to characterize the hospital-at-home service in the framework of CHS in the southern region of Israel, and to evaluate its effect on the number of hospitalizations, the number of hospitalization days, the number of emergency room visits, and the cost of hospitalization.

## Methods

### Study population

The present study was based on data collected from the computerized medical records of the patients who were treated in the home care unit for at least one month between the years 2013–2020.

### Data collection

The data collected included sociodemographic information (age, sex, family status, information on the primary caregiver), medical condition, and the results of the geriatric assessments that the patients underwent including co-morbidity, regular medications, disease burden (Charlson's comorbidity index [CCI]) [21], and the Cumulative Illness Rating Scale-Geriatric (G-CIRS) [22]. The cognitive state was evaluated with the Mini-Mental State Examination (MMSE) [23], mood assessed by the Patient Health Questionnaire-2 (PHQ-2) [24] and anxiety assessed by the General Anxiety Disorder questionnaire (GAD-7) [25]. The diagnoses of dementia, depression, and anxiety were based on the Diagnostic and Statistical Manual of Mental Disorders, Fifth Edition (DSM 5) criteria [26]. The Norton questionnaire [27] was used to estimate the risk to develop pressure ulcers, the Barthel Index [28] and the Older Americans Resources and Service Instrumental Activity of Daily Living (OARS-IADL) [29] were used to assess functional state, and the Mini-Nutritional Assessment Short Form (MNA-SF) [30] to assess nutritional state. In addition, data were collected on the number of hospitalizations and hospitalization days, the number of emergency room visits, the cost of hospitalization, and the cost of employing the hospital-at-home team.

Four outcome measures were assessed in the study: (1) the number of hospitalizations, (2) the number of hospitalization days, (3) the number of emergency room (ER) visits, and (4) the cost of hospitalizations, The intent was to assess these parameters in terms of in-hospital hospitalization only.

All variables were calculated as mean per patient per month and compared among three periods of time: the six months prior to admission to the unit, the treatment period in the unit, and the six months following discharge from the unit.

### Statistical analyses

Statistical analyses were conducted using SPSS version 26. Categorical variables (mortality data, family status, sex, et al.) are presented as frequencies and percentages. Quantitative variables (age, cognitive function, hospitalization cost, et al.) are presented as mean ± standard deviation (SD) and median. Bivariate analyses were performed to compare the dependent variables (number of hospitalizations, number of hospital days, number of emergency room visits, and hospitalization cost) for the three study periods. All dependent variables are presented as mean ± standard deviation (SD) and median per patient per month. The comparison among periods was conducted by non-parametric tests for two related samples, due to their non-normal distribution. Linear regression models were developed to predict changes in the study outcomes (length of hospitalization, number of hospitalizations, number of emergency room visits and hospitalization costs) over the course of treatment. The models were built for the entire study population and for subgroups of patients. All models included: gender, family status, source of referral, dementia, geriatric syndromes, deconditioning, CHF, mechanical ventilation, acute infection, COPD, malignances, other diseases, CCI, and CIRS-G.

Hospitalization costs were presented in United States dollars (USD), according to the conversion rate on 5/19/2023, which was 3.645 shekels per dollar. Statistical significance was set at P < 0.05.

The ethics committee of CHS approved the study (approval #0073-15-COM2) and exempted it from the need to obtain informed consent.

## Results

Overall, 678 patients were treated in the hospital-at-home service unit from 2013 to 2020. Of these, 623 who were treated for at least one month were included in the study. The mean age was 83.7 ± 9.2 years and most were men (N = 366, 58.7%). Most of the patients (79.1%) had been hospitalized at least once over the 6-month period prior to their admission to the unit, and this was one of the main reasons for referring them to the service.

Almost all (84.6%) were referred to the unit from the community by family physicians. The most common reasons for admission to the unit were geriatric syndromes (56.7%), acute functional decline (deconditioning) (21.2%), and severe heart failure (12%). Most of the patients had an impaired cognitive state with a mean MMSE score of 12 ± 10.2, an impaired functional state with a mean Barthel Index score of 23.9 ± 25.1, and a high burden of disease with a mean Charlson's  comorbidity index total combined score of 7.7 ± 2.3 (Table 1). The study population was divided into three groups: 86 (13.8%) patients who were treated in the unit until the end of the study follow-up period and were not discharged, were included in Group 1; 142 (22.8%) patients who were treated in the unit and died during the course of the treatment, were included in Group 2; and 395 (63.4%) patients who were treated in the unit and were discharged after their condition stabilized, were included in Group 3. Of the 395 patients who were discharged at the end of treatment, 306 (77.5%) survived for more than six months. Table 2 shows a comparison of the characteristics of these three groups.

**Table 1:** Characteristics of the study population (N = 623)

Variable	Result
*Age*	
Mean ± SD	83.7 ± 9.2
Median	85
Range	25–109
*Sex [N (%)]*	
Male	366 (58.7)
Female	257 (41.3)
*Family status [N (%)]*	
Single	2 (0.3)
Divorced	22 (3.5)
Widowed	343 (55.1)
Married	256 (41.1)
*Source of referral [N (%)]*	
Community	527 (84.6)
Hospital	96 (15.4)
*MMSE*	
Mean ± SD	12.0 ± 10.2
Median	14
Range	0–30
Missing	269
*PHQ-2*	
Mean ± SD	2.3 ± 3.9
Median	0
Range	0–6
Missing	421
*BADL*	
Mean ± SD	23.9 ± 25.1
Median	15
Range	0–95
Missing	180
*IADL*	
Mean ± SD	1.2 ± 2.0
Median	0
Range	0–11
Missing	185
*Norton*	
Mean ± SD	11.1 ± 3.1
Median	11
Range	3–19
Missing	197
*MNA*	
Mean ± SD	6.6 ± 3.0
Median	6
Range	1–24
Missing	269
*CCI*	
Mean ± SD	3.7 ± 2.2
Median	3
Range	0–14
Missing	197
*CIRS-G total score*	
Mean ± SD	14.98 ± 6.96
Median	15
Range	0–32
Missing	268
*Number of hospitalizations prior to admission to unit [N (%)]*	
0	130 (20.9)
1	179 (28.7)
2	146 (23.4)
3	85 (13.6)
4	46 (7.4)
5	15 (2.4)
6	9 (1.4)
7+	13 (2.1)
*Reasons for admission to the Hospital-at-Home Unit [N(%)]*	
Geriatric syndromes (pressure sores, dementia, BPSD, malnutrition, delirium, etc.)	619 (56.7)
Deconditioning	231 (21.2)
Chronic heart failure (NYHA 3–4 class)	131 (12.0)
COPD	46 (4.2)
Active cellulitis/UTI/pneumonia	42 (3.8)
Mechanical ventilation	12 (1.1)
Neoplasm	5 (0.5)
Other	5 (0.5)
*Treatment pattern [N (%)]*	
Treated in unit and discharged due to end of treatment	395 (63.4)
Treated in unit and died during treatment	142 (22.8)
Treated in unit until the end of the treatment period	86 (13.8)
*Mortality following discharge from the unit [N (%)]*	
Survived for 6 months after discharge	306 (77.5)
Died during the six months following discharge from the unit	89 (22.5)

*MMSE* mini-mental state examination, *BADL* Barthel Index, *IADL* instrumental activity of daily living, *MNA* mini-nutritional assessment, *PHQ-2* Patient Health Questionnaire-2, *CCI* Charlson Comorbidity Index, *BPSD* behavioral and psychological symptoms of dementia, *PEG* percutaneous endoscopic gastrostomy, *NGT* nasogastric tube, *NYHA* New York Heart Association, *COPD* chronic obstructive pulmonary disease, *UTI* urinary tract infection

**Table 2:** Comparison of the characteristics of the patients by study group

	*Group 1 (N = 86)	†Group 2 (N = 142)	‡Group 3 (N = 395)	§p-value	‖p-value	**p-value
*Age*						
Mean ± SD	80.8 ± 12.2	85.3 ± 9.8	83.7 ± 8.1			
Median	83.5	86	85	0.002	0.01	0.08
Range	27–109	25–96	46–105			
*Indication for admission to the unit (more than one response is possible) [N (%)]*						
Dementia	33 (38.4)	75 (52.8)	166 (42)	0.03	0.53	0.03
Other geriatric syndromes	52 (60.5)	90 (63.4)	203 (51.4)	0.7	0.13	0.01
Deconditioning	30 (34.9)	41 (28.9)	160 (40.5)	0.3	0.34	0.01
Acute inflammation	10 (11.6)	5 (3.5)	27 (6.8)	0.02	0.13	0.15
Chronic heart failure	27 (31.4)	25 (17.6)	79 (20)	0.02	0.02	0.54
Mechanical ventilation	5 (5.8)	4 (2.8)	3 (0.8)	0.26	< 0.001	0.06
*MMSE*						
Mean ± SD	10.9 ± 11.9	7.6 ± 9.6	13.6 ± 9.6			
Median	5.5	2	15	0.09	0.07	< 0.001
Range	0–30	0–30	0–30			
Missing	28	73	168			
*BADL*						
Mean ± SD	20.6 ± 26.1	11.8 ± 18.5	28.2 ± 25.4			
Median	5	5	25	0.02	0.03	< 0.001
Range	0–70	0–70	0–95			
Missing	19	57	104			
*CCI*						
Mean ± SD	3.6 ± 2	3.2 ± 2	3.8 ± 2.3			
Median	3	3	3	0.3	0.4	0.02
Range	1–9	1–11	0–14			
Missing	21	65	111			
*CIRS-G severity index*						
Mean ± SD	2.7 ± 0.8	2.6 ± 0.9	2.4 ± 1			
Median	15	16	15.5	0.5	0.1	0.3
Range	0.3–4.5	0.3–4	0.6–6.7			
Missing	43	87	42			
*Number of drugs on admission*						
Mean ± SD	7.8 ± 4.8	7.1 ± 3.7	8.2 ± 4.1			
Median	7	7	8	0.2	0.5	0.004
Range	0–20	0–18	0–22			
*Norton*						
Mean ± SD	10.9 ± 3.8	9.6 ± 3.8	11.5 ± 3.3	0.03	0.2	< 0.001
Median	11	9	12			
Range	4–18	3–17	5–19			
Missing	21	64	112			
*MNA*						
Mean ± SD	6.8 ± 3.1	5.8 ± 2.7	6.8 ± 3.04			
Median	7	5.5	7	0.03	1	0.004
Range	0–14	1–14	0–14			
Missing	22	64	114			

*MMSE* mini-mental state examination, *BADL* Barthel Index, *MNA* Mini-nutritional assessment, *CCI* Charlson Comorbidity Index, *CIRS-G* Cumulative Illness Rating Scale-Geriatric

*Group 1—treated in the unit until the end of the treatment period

^†^Group 2—treated in the unit and died during the treatment period

^‡^Group 3—treated in the unit and discharged due to completion of treatment

^§^Group 1 vs. Group 2

‖Group 1 vs. Group 3

**Group 2 vs. Group 3

### Characteristics of the treatment course

Overall, the mean length of stay in the unit was 7.8 months. This period decreased from 13.2 ± 12.01 months in 2013 to 2.9 ± 2.2 months in 2020. The mean monthly number of patients increased consistently over the years. In 2020 (the final year of the study) we treated 94 patients monthly.

Over the course of treatment in the unit the mean number of visits per patient was 2.3 ± 1.9 per month for doctors, 2.0 ± 1.8 per month for nurses, 1.5 ± 2.5 for physical therapists, 0.4 ± 0.5 for dieticians and 0.4 ± 0.4 for social workers. The most common treatment interventions were change in medications (70%), nutritional counseling (65%), physical therapy (44.5%), treatment for pressure ulcers (39.2%), treatment for dementia-related behavioral disturbances (32.1%), and control of pain (30.5%).

### Outcomes of treatment in the unit

For the entire study population there was statistically significant decrease in the number of hospitalization days (2.84 ± 4.35 before admission vs. 1.70 ± 3.80 during treatment, p < 0.001). In addition, there was a non-significant trend towards a decrease in the number of hospitalizations (p = 0.082) and the number of emergency room visits (p = 0.059). When analyzing the three groups separately, in Group 1 there was a statistically significant decrease in the number of hospitalizations (0.37 ± 0.35 before admission vs. 0.16 ± 0.28 during treatment, p < 0.001), and in the number of hospitalization days (4.50 ± 6.58 before admission vs. 0.96 ± 1.81 during treatment, p < 0.001). In Group 2 there were no significant differences in any of the outcomes. In Group 3 there was a statistically significant decrease in the number of hospitalizations (0.28 ± 0.24 before admission vs 0.25 ± 0.5 six months following discharge from the unit, p < 0.001), in the number of hospitalization days (2.38 ± 3.39 before admission vs. 1.59 ± 4.17 during treatment, p = 0.004). which remained statistically significant six months after discharge from the unit (2.38 ± 3.39, p < 0.001), and in the number of emergency room visits (0.09 ± 0.15 before admission vs. 0.06 ± 0.24 during treatment, p = 0.042) which remained statistically significant six months after discharge from the unit (0.05 ± 0.12, p < 0.001) (Table 3).

**Table 3:** Main outcomes by groups and time periods

	N	Six months prior to admission to the unit	During treatment in the unit	Six months after discharge from the unit	§p-value	‖p-value	**p-value
*Number of hospitalizations*							
All patients	623						
Mean ± SD		0.31 ± 0.3	0.27 ± 0.51	–	0.08	–	–
Median		0.33	0.05	–			
*Group 1	86						
Mean ± SD		0.37 ± 0.35	0.16 ± 0.28	–	< 0.001	–	–
Median		0.33	0.04	–			
†Group 2	142						
Mean ± SD		0.35 ± 0.41	0.4 ± 0.59	–	0.36	–	–
Median		0.33	0.19	–			
‡Group 3	395						
Mean ± SD		0.28 ± 0.24	0.25 ± 0.5	0.24 ± 0.59	0.19	< 0.001	0.6
Median		0.17	0	0			
*Number of hospitalization days*							
All patients	623						
Mean ± SD		2.84 ± 4.35	1.7 ± 3.8	–	< 0.001	–	–
Median		1.5	0.16	–			
*Group 1	86						
Mean ± SD		4.5 ± 6.58	0.96 ± 1.81	–	< 0.001	–	–
Median		1.75	0.16	–			
†Group 2	142						
Mean ± SD		3.12 ± 4.79	2.46 ± 3.41	–	0.19	–	–
Median		1.67	0.86	–			
‡Group 3	395						
Mean ± SD		2.38 ± 3.39	1.59 ± 4.17	1.73 ± 4.17	0.004	< 0.001	0.6
Median		1.33	0	0			
*Number of emergency room visits*							
All patients	623						
Mean ± SD		0.08 ± 0.14	0.06 ± 0.22	–	0.06	–	–
Median		0	0	–			
*Group 1	86						
Mean ± SD		0.07 ± 0.14	0.05 ± 0.13	–	0.42	–	–
Median		0	0	–			
†Group 2	142						
Mean ± SD		0.06 ± 0.12	0.06 ± 0.22	–	0.9	–	–
Median		0	0	–			
‡Group 3	395						
Mean ± SD		0.09 ± 0.15	0.06 ± 0.24	0.05 ± 0.12	0.04	< 0.001	0.7
Median		0	0	0			

*Group 1—treated in the unit until the end of the treatment period

^†^Group 2—treated in the unit and died during the treatment

^‡^Group 3—treated in the unit and discharged due to completion of treatment

^§^Comparison between the six months prior to admission to the unit and the period of treatment

‖Comparison between the six months prior to admission to the unit and the period of six months following discharge from the unit

**Comparison between the period of treatment in the unit and the period of six months following discharge from the unit

### Hospitalization costs

Table 4 presents the changes in hospitalization costs over the different time periods for the entire study population and each of the three study groups separately. For the entire study population there was a statistically significant decrease of 33.6% in the cost of hospitalization (1606 ± 2170 USD before admission vs 1066 ± 2082 USD during treatment, P < 0.001), which remained statistically significant after adjustment for the cost of employing the staff. In Group 1 there was a statistically significant decrease in the cost of hospitalization between the six months prior to admission and the time during treatment in the unit (2417 ± 3209 USD vs. 705 ± 1419 USD, P < 0.001). In Group 3 there was a statistically significant decrease in the cost of hospitalization between the six months prior to admission and the time during treatment in the unit (1341 ± 1659 USD vs. 939 ± 2267 USD, P < 0.001), which remained statistically significant six months after discharge from the unit (983 ± 3046 USD, P < 0.001). After adding the cost of employing the unit staff, a statistically significant decrease in the cost of hospitalization was found in Group 1 only.

**Table 4:** Comparison of the hospitalization costs between all groups of the patients

Group	N	Monthly hospitalization cost per patient, USD			§p-value	‖p-value	**p-value
		Six months prior to admission to the unit	During treatment in the unit	Six months after discharge from the unit			
All patients	623			–	< 0.001	–	–
Mean ± SD		1606 ± 2170	1066 ± 2082				
Median		946	120				
*Group 1	86			–	< 0.001	–	–
Mean ± SD		2417 ± 3209	705 ± 1419				
Median		1314	129				
†Group 2	142			–	0.44	–	–
Mean ± SD		1850 ± 2494	1638 ± 2225				
Median		1160	663				
‡Group 3	395				0.003	< 0.001	0.5
Mean ± SD		1341 ± 1659	939 ± 2267	983 ± 3046			
Median		852	0	0			

*Group 1—treated in the unit until the end of the treatment period

^†^Group 2—treated in the unit and died during the treatment

^‡^Group 3—treated in the unit and discharged due to completion of treatment

^§^Comparison between the six months prior to admission to the unit and the period of treatment

^‖^Comparison between the six months prior to admission to the unit and the period of six months following discharge from the unit

**Comparison between the period of treatment in the unit and the period of six months following discharge from the unit

### Multivariate models

Several prediction models were assessed for change in hospitalization days, change in number of hospitalizations, change in number of emergency room visits, and change in hospitalization cost. The models were built for the entire study population and for subgroups of patients. The only variable that predicted a decrease in the number of hospitalization days was deconditioning (B = −0.179, P = 0.006). A decrease in the number of emergency room visits was predicted by geriatric syndromes only (B = −0.169, P = 0.004) and a decrease in the hospitalization cost was predicted only by deconditioning (B = −0.177, P = 0.006). In subgroup analyses, a decrease in the number of days of hospitalization was predicted only by the diagnosis of mechanical ventilation in the group of patients who died during hospitalization (Group 2) and by deconditioning in the group of patients who were discharged due to completion of treatment (Group 3). A decrease in the number of emergency room visits was predicted by the diagnosis of geriatric syndromes only (Group 3), and a decrease in the hospitalization cost was predicted by the diagnosis of mechanical ventilation (Group 2), and the diagnosis of deconditioning (Group 3) only (Table 5). There was no association between the burden of disease as measured by CCI and G-CIRS and the outcome of treatment.

**Table 5:** Linear regression model to predict changes in study outcomes over the course of treatment

	Entire study sample	Subgroup analysis		
	(N = 623)	*Group 1 (N = 86)	†Group 2 (N = 142)	‡Group 3 (N = 395)
Hospitalization days (N)	Deconditioning (B = −0.18, P = 0.006) R2 = 0.097	Non-significant	Mechanical ventilation (B = −0.4, p = 0.007) R2 = 0.253	Deconditioning (B = -0.24, p = 0.003) R2 = 0.103
Emergency room visits (N)	Geriatric syndromes (B = -0.17, P = 0.004) R2 = 0.056	Non-significant	COPD (B = 0.36, p = 0.044) R2 = 0.218	Geriatric syndromes (B = -0.16, P = 0.025) R2 = 0.06
Hospitalization cost	Deconditioning (B = -0.18, P = 0.006) R2 = 0.096	Non-significant	Mechanical ventilation (B = −0.35, p = 0.017) R2 = 0.232	Deconditioning (B = -0.26, p = 0.001) R2 = 0.116
Hospitalizations (N)	Non-significant	Non-significant	Non-significant	Non-significant

All models included: gender, family status, source of referral, dementia, geriatric syndromes, deconditioning, CHF, mechanical ventilation, acute infection, COPD, malignances, other diseases, CCI and CIRS-G

*Group 1—treated in the unit until the end of the treatment period

^†^Group 2—treated in the unit and died during the treatment period

^‡^Group 3—treated in the unit and discharged due to completion of treatment

## Discussion

The results of this study indicate that the current model of hospital-at-home service, which was meant to provide a solution for homebound patients with a broad range of medical problems, had a positive effect on the utilization of healthcare services. A decrease in the number of days of hospitalization and the cost of hospitalization was observed, and the decrease in the cost of hospitalization remained statistically significant even after adjusting for the cost of staff employment. These results support and encourage implementation of the current model as an alternative to hospitalization in a general hospital. The findings of the current study are consistent with those of previous studies that showed the benefits of a home care unit as an alternative to hospitalization, especially relating to care for medical conditions such as heart failure, COPD, infections, pneumonia, following acute stroke [4–6, 8, 9, 11], and in a mixed geriatric population with a high burden of disease [17]. The characteristics of the current model that were like the characteristics of other successful models of hospital-at-home services included a multidisciplinary team, continuous contact with patients, and unlimited treatment time [5, 7, 31–33]. The unique elements of the current model are a geriatric team that includes a geriatrician and a nurse with experience in geriatrics, the substantial involvement of other health professionals (physiotherapist and occupational therapist, dietitian, and social worker), the determination of very specific treatment goals, and periodic team meetings to discuss continuation or termination of treatment.

The patients who gained the greatest benefit were those who continued treatment in the unit and were not sent back to the primary care clinic. Among the patients who were sent back to the primary care clinic there was an increase in the number of hospitalizations, in the number of hospital days, and in the cost of hospitalization, although to a lesser degree than before admission to the unit. There were no changes in any treatment parameter among patients who died over the course of treatment in the unit. The patients in this group were older, with more significant cognitive and functional impairment, and suffered more from dementia and/or geriatric syndromes. It might be reasonable to define their condition as terminal and requiring palliative care, which has been shown to be effective in preventing suffering and improving the quality of life of the patients and their close relatives [34, 35], but not necessarily effective in terms of the rate of hospitalizations or cost of service [16, 34].

The length of stay in the unit has shortened over the years, as the staff has gained experience, and decreased from 13.22 ± 12.01 months in 2013 to 2.99 ± 2.23 months in 2020, without a negative effect on treatment outcomes. Over the course of treatment most of the patients needed visits from the entire staff, emphasizing the importance of a multidisciplinary team in the treatment of complex homebound patients.

The multivariate analysis showed that patients who were admitted for deconditioning or geriatric syndromes had a greater chance of reduction in the number of hospitalization days, the number of emergency room visits, and the cost of hospitalization, probably because of the treatment provided in the hospital-at-home service.

The composition of the patient groups and the way the service was implemented did not change during the Corona pandemic. Furthermore, since the Corona period there has been a higher rate of patient willingness to be treated in the home setting, thus avoiding hospitalization.

### Strengths and limitations

The unique sample of homebound older patients with significant functional and cognitive impairment, including a high rate of dementia and geriatric syndromes, along with the presence of a multidisciplinary geriatric team are the strengths of the study. Detailed information about the patients, including details of geriatric syndromes, the results of the comprehensive geriatric assessment, the scope of the team's activities, and the types of interventions are additional strengths.

The study limitations were its retrospective design and lack of a control group. To overcome this obstacle, we compared 3 different periods of time (the period of six months before admission to the unit, the period of treatment in the unit, and the period of six months following discharge from the unit) for every study outcome. Another limitation was missing data for some of the variables. To neutralize the effect of missing data on the study outcomes, we conducted an additional statistical analysis on the group of patients without any missing data, but this analysis did not change the study conclusions. The comprehensive geriatric assessment that was conducted for all patients might have had an effect on the number of hospitalizations, since there is a known direct association between this assessment and reduction in hospitalizations in the adult population [36]. Another important limitation is the absence of data on the quality of life of the patients and their family members. The routine assessment of patients, over the course of treatment, did not include quality of life assessment and, due to the retrospective nature of the study, we could not add this element. Based on existing data in the literature [7–10, 12, 34, 35, 37], patients’ quality of life was improved during home care treatment in a broad spectrum of home hospitalization programs. The high level of availability of the staff, including availability by telephone and the fact that the patients remained at home as opposed to hospitalization, made a substantial contribution to this improvement in their quality of life. The investigators observed that most patients expressed a high level of satisfaction with the service. Unfortunately, this important information is missing in the present study and should be tested in future studies. Another limitation was the inability to prove a causal relationship between the intervention and economic outcomes. It was not possible in the present study to conduct a propensity score or difference in differences analysis. The investigators are planning to study this issue in the next study, which will be designed to compare between hospital-at-home patients from the present study and those patients who underwent geriatric assessment at home but were not admitted to the hospital-at-home service.

## Conclusions and implications

One of the complex challenges that the healthcare system faces is the accelerated aging of the population and the increase in the number of homebound patients with medical complexity.

The findings of the present study support the conclusion that treatment provided by a multidisciplinary team led by a geriatrician, within the framework of a hospital-at-home service for homebound adults with a high burden of disease and an increased risk of hospitalization, can lead to a significant reduction in the number of hospitalizations and their cost. The results of the present study, like previous ones, present an alternative to the “traditional” structure of the healthcare system, which can provide a solution to the present challenge. The investigators recommend integration of the recommended model into the healthcare system with the aim of enhancing the provision of a high-level medical solution, which also is economically efficient, at the patient’s home.

## Data Availability

Clalit Health Services does not allow sharing the data.

## Abbreviations

HMO's: Health Maintenance Organizations
CHS: Clalit Health Services
BPSD: Behavioral and psychological
ER: Emergency room
CCI: Charlson's comorbidity index
G-CIRS: Cumulative Illness Rating Scale-Geriatric
MMSE: Mini-Mental State Examination
PHQ-2: Patient Health Questionnaire-2
GAD-7: General Anxiety Disorder questionnaire
BADL: Barthel Index
OARS-IADL: Older Americans Resources and Service Instrumental Activity of Daily Living
IADL: Instrumental Activity of Daily Living
MNA-SF: Mini-Nutritional Assessment Short Form
PEG: Percutaneous endoscopic gastrostomy
NGT: Nasogastric tube
NYHA: New York Heart Association
COPD: Chronic obstructive pulmonary disease
UTI: Urinary tract infection
